# 2,5-Dimethyl­pyrazine 1,4-dioxide

**DOI:** 10.1107/S1600536809046741

**Published:** 2009-11-11

**Authors:** Carlton J. Brown, Jacqueline M. Knaust

**Affiliations:** a Allegheny College, Chemistry Department, 520 North Main St., Meadville, PA 16335, USA

## Abstract

The title compound, C_6_H_8_N_2_O_2_, was prepared from 2,5-dimethyl­pyrazine, acetic acid, and hydrogen peroxide. The 2,5-dimethyl­pyrazine 1,4-dioxide mol­ecule is located on an inversion center. π–π inter­actions between neighboring 2,5-dimethyl­pyrazine 1,4-dioxide mol­ecules are observed with an inter­planar distance of 3.191 Å. Each 2,5-dimethyl­pyrazine 1,4-dioxide mol­ecule is linked to four neighboring *N*-oxide mol­ecules through C—H⋯O hydrogen-bonding inter­actions, forming two-dimensional layers.

## Related literature

For the synthesis of 2,2′-bipyridine *N,N*′-dioxide, see: Simpson *et al.* (1963 ▶). For the synthesis of lanthanide coordination networks with pyrazine *N,N*′-dioxide, see: Cardoso *et al.* (2001 ▶); Sun *et al.* (2004 ▶). For the use of 2,5-dimethyl­pyrazine 1,4-dioxide in the synthesis of transition metal coordination networks, see: Shi, Sun *et al.* (2006 ▶); Shi, Zhang *et al.* (2006 ▶); Shi *et al.* (2007 ▶); Sun, Gao *et al.* (2005 ▶); Sun, Wang *et al.* (2005 ▶). For related structures, see: Näther *et al.* (2002 ▶); Gratton & Knaust (2009 ▶).
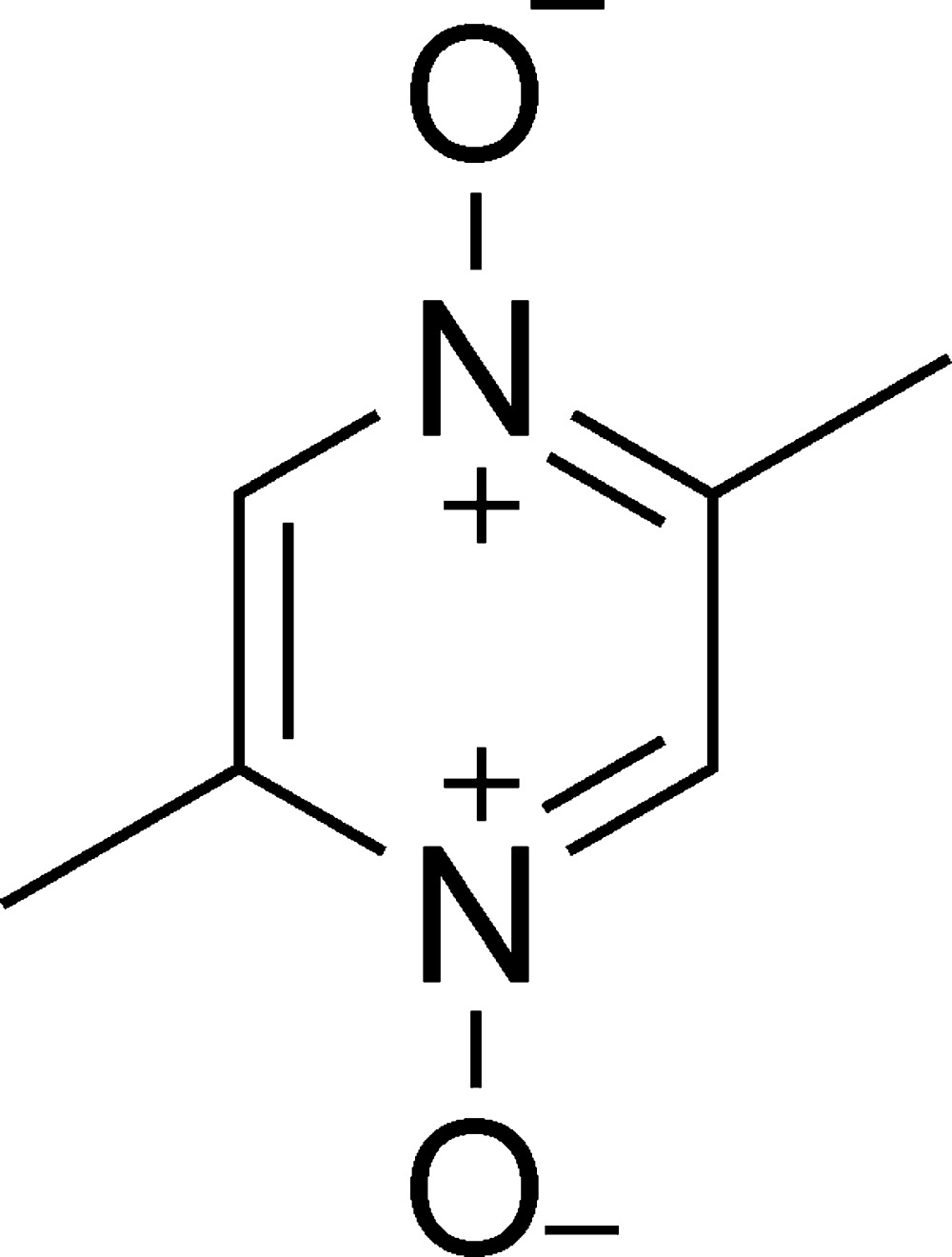


## Experimental

### 

#### Crystal data

C_6_H_8_N_2_O_2_
*M* *_r_* = 140.14
Monoclinic, 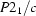

*a* = 3.9971 (8) Å
*b* = 8.9176 (17) Å
*c* = 8.9249 (17) Å
β = 102.205 (3)°
*V* = 310.93 (10) Å^3^
*Z* = 2
Mo *K*α radiation
μ = 0.12 mm^−1^
*T* = 173 K
0.45 × 0.12 × 0.11 mm

#### Data collection

Bruker SMART APEX CCD diffractometer
Absorption correction: multi-scan (*SADABS*; Bruker, 2001 ▶) *T*_min_ = 0.623, *T*_max_ = 0.746
2388 measured reflections
965 independent reflections
811 reflections with *I* > 2σ(*I*)
*R*_int_ = 0.023

#### Refinement

*R*[*F* ^2^ > 2σ(*F* ^2^)] = 0.049
*wR*(*F* ^2^) = 0.144
*S* = 1.07
965 reflections
47 parameters
H-atom parameters constrained
Δρ_max_ = 0.62 e Å^−3^
Δρ_min_ = −0.34 e Å^−3^

### 

Data collection: *SMART* (Bruker, 2007 ▶); cell refinement: *SAINT-Plus* (Bruker, 2007 ▶); data reduction: *SAINT-Plus*; program(s) used to solve structure: *SHELXS97* (Sheldrick, 2008 ▶); program(s) used to refine structure: *SHELXL97* (Sheldrick, 2008 ▶); molecular graphics: *X-SEED* (Barbour, 2001 ▶); software used to prepare material for publication: *X-SEED*.

## Supplementary Material

Crystal structure: contains datablocks I, global. DOI: 10.1107/S1600536809046741/zl2251sup1.cif

Structure factors: contains datablocks I. DOI: 10.1107/S1600536809046741/zl2251Isup2.hkl

Additional supplementary materials:  crystallographic information; 3D view; checkCIF report

## Figures and Tables

**Table 1:** Hydrogen-bond geometry (Å, °)

*D*—H⋯*A*	*D*—H	H⋯*A*	*D*⋯*A*	*D*—H⋯*A*
C1—H1*C*⋯O1^i^	0.98	2.41	3.3290 (15)	155
C3—H3⋯O1^i^	0.95	2.31	3.1863 (15)	153

Symmetry code: (i) 
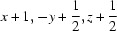
.

**Fig. 1.**
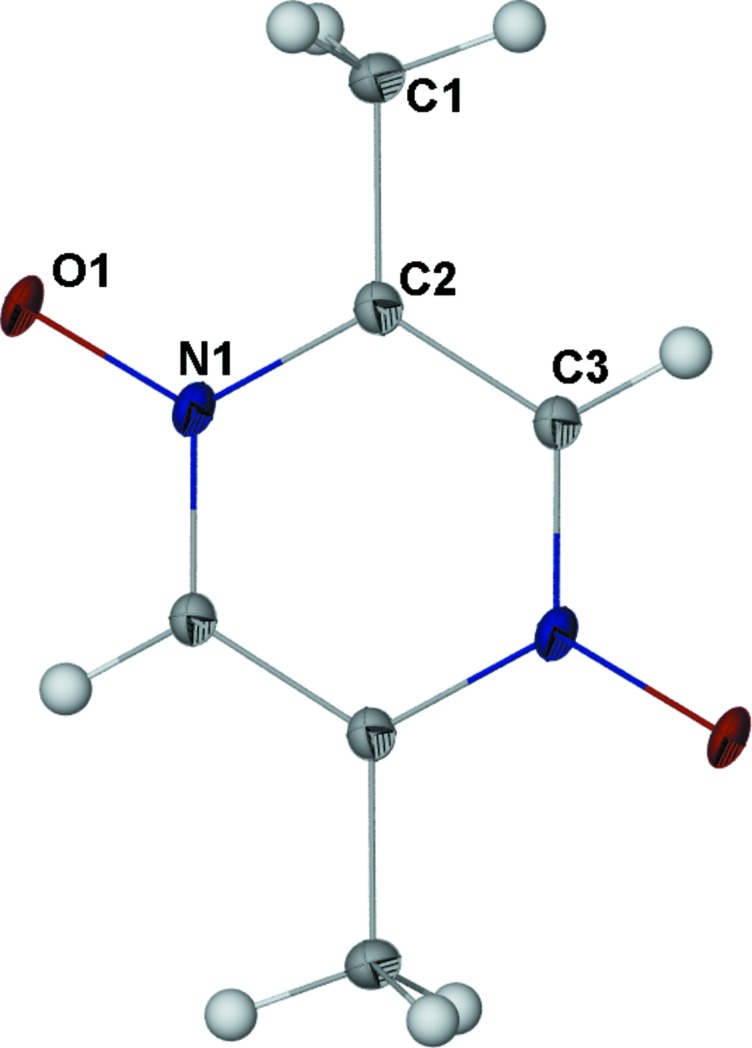
The molecular structure of the title compound with atom labels and 50% probability displacement ellipsoids for non-H atoms. Atoms not labeled are generated by the symmetry operator (i) -x + 1,-y, -z + 1.

**Fig. 2.**
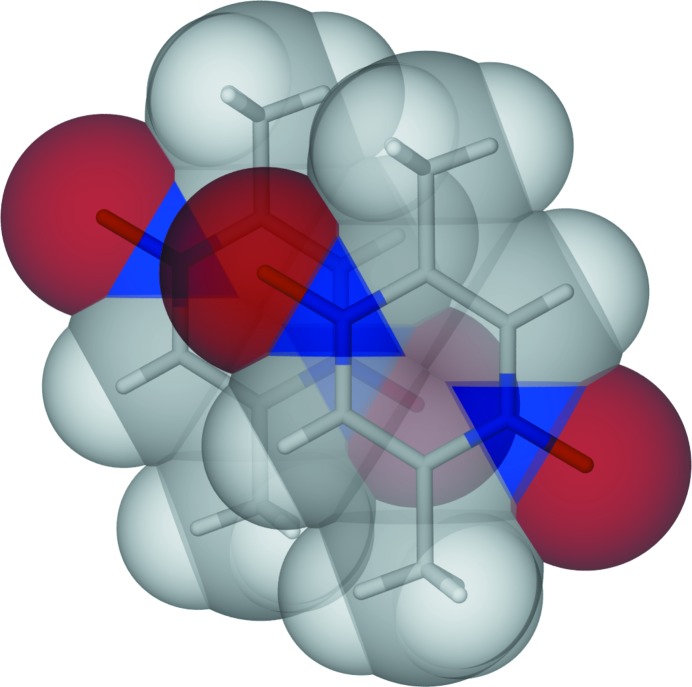
Space filling representation of the π-cloud interactions between neighboring 2,5-dimethylpyrazine 1,4-dioxide molecules.

**Fig. 3.**
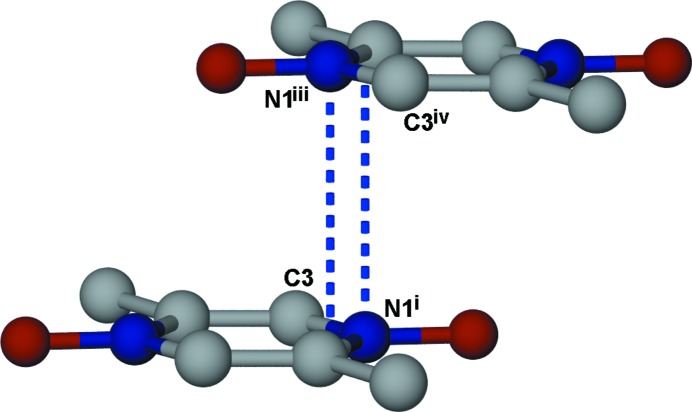
Ball and stick representation of the π-cloud interactions between neighboring 2,5-diethylpyrazine 1,4-dioxide molecules. symmetry codes: (i) -x + 1, -y, -z + 1; (iii) x + 1, y, z; (iv) -x + 2, -y, -z + 1

**Fig. 4.**
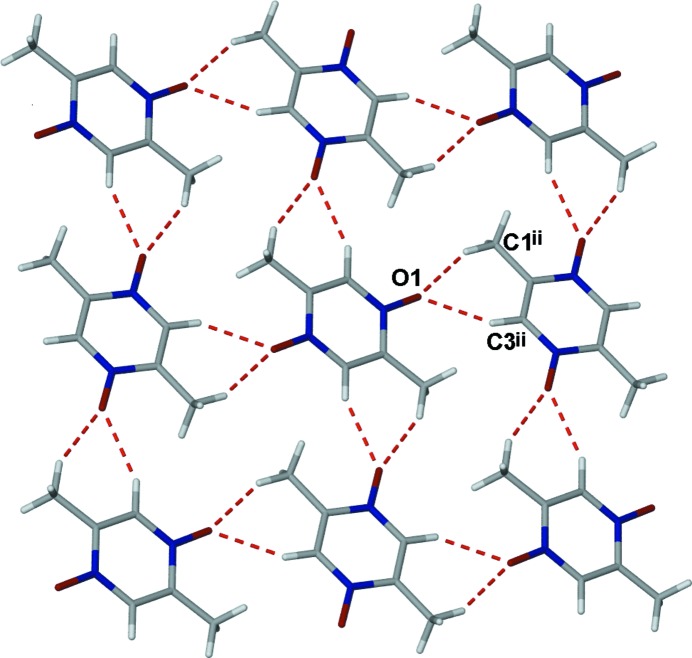
C—H···O hydrogen bonding interactions between neighboring 2,5-dimethylpyrazine 1,4-dioxide molecules. Hydrogen bonds are shown as dashed lines. Symmetry code: (ii) x + 1, -y + 1/2, z +1 /2.

**Fig. 5.**
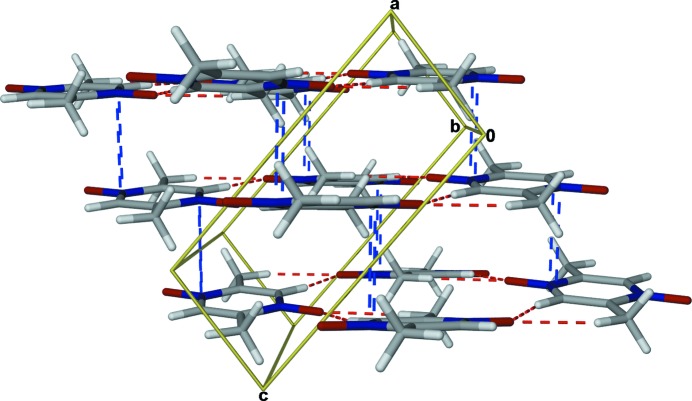
Packing of the title compound viewed down the b axis. Hydrogen bonds are shown as dashed red lines, and π-cloud interactions are shown as dashed blue lines.

## supplementary crystallographic information

### Comment

The use of pyrazine *N,N*'-dioxide in the synthesis of lanthanide coordination networks has been of recent interest (Cardoso *et al.* (2001), and Sun *et al.* (2004)). Shi, Sun *et al.* (2006), Shi, Zhang *et al.* (2006), Shi *et al.* (2007), Sun, Gao *et al.* (2005), and Sun, Wang *et al.* (2005) recently reported the use 2,5-dimethylpyrazine 1,4-dioxide in the synthesis of a transition metal coordination networks. The title compound was prepared using the reaction conditions described by Simpson *et al.* (1963) to prepare 2,2'-bipyridine *N,N*'-dioxide.

The asymmetric unit of the title compound contains half of a 2,5-dimethylpyrazine 1,4-dioxide molecule (Figure 1) and the N-oxide molecule lies on an inversion center. π-Cloud interactions between neighboring 2,5-dimethylpyrazine 1,4-dioxide molecules are observed with an interplanar distance of 3.191 Å (Figure 2); there is a slippage of 2.408 Å such that N1^iii^ on the neighboring N-oxide molecule lies directly over the centroid of the C3—N1^i^ bond [symmetry codes: (i) -*x* + 1, -*y*, -*z *+ 1; (iii) *x* + 1, *y*, *z*] (Figure 3). The title compound forms eight C—H···O hydrogen bonds with four neighboring N-oxide molecules, and these hydrogen bonding interactions result in the formation of two-dimensional layers (Figure 5); whereas in the related structures of 2-methylpyrazine 1,4-dioxide and pyrazine N,N'-dioxide, the N-oxide molecules form hydrogen bonded ribbons and a three-dimensional network, respectively (Gratton *et al.* (2009), Näther *et al.* (2002)). A packing diagram of the title compound is given in Figure 5.

### Experimental

2,5-Dimethylpyrazine (6.99 ml, 64.0 mmol), acetic acid (75 ml), and 30% hydrogen peroxide (13 ml) were heated at 343–353 K for 3 h. Additional hydrogen peroxide (9 ml) was added, and heating was continued. After an additional 19 h of heating the solution was cooled to room temperature. Crystals formed upon the addition of acetone (1*L*) and cooling to 273 K, and were recrystallized from hot water by addition of excess acetone and cooling to 273 K.

### Refinement

All H atoms were positioned geometrically and refined using a riding model with C—H = 0.95–0.99 Å and with *U_iso_*(H) = 1.2 (1.5 for methyl groups) times *U*_eq_(C).

### Crystal data

**Table:**

C_6_H_8_N_2_O_2_	*F*(000) = 148
*M**_r_* = 140.14	*D*_x_ = 1.497 Mg m^−^^3^
Monoclinic, *P*2_1_/*c*	Mo *K*α radiation, λ = 0.71073 Å
Hall symbol: -P 2ybc	Cell parameters from 958 reflections
*a* = 3.9971 (8) Å	θ = 3.3–31.5°
*b* = 8.9176 (17) Å	µ = 0.12 mm^−^^1^
*c* = 8.9249 (17) Å	*T* = 173 K
β = 102.205 (3)°	Rod, colorless
*V* = 310.93 (10) Å^3^	0.45 × 0.12 × 0.11 mm
*Z* = 2	

### Data collection

**Table:**

Bruker SMART APEX CCD diffractometer	965 independent reflections
Radiation source: fine-focus sealed tube	811 reflections with *I* > 2σ(*I*)
graphite	*R*_int_ = 0.023
ω scans	θ_max_ = 31.5°, θ_min_ = 3.3°
Absorption correction: multi-scan (*SADABS*; Bruker, 2001)	*h* = −5→5
*T*_min_ = 0.623, *T*_max_ = 0.746	*k* = −12→9
2388 measured reflections	*l* = −12→8

### Refinement

**Table:**

Refinement on *F*^2^	Primary atom site location: structure-invariant direct methods
Least-squares matrix: full	Secondary atom site location: difference Fourier map
*R*[*F*^2^ > 2σ(*F*^2^)] = 0.049	Hydrogen site location: inferred from neighbouring sites
*wR*(*F*^2^) = 0.144	H-atom parameters constrained
*S* = 1.07	*w* = 1/[σ^2^(*F*_o_^2^) + (0.0934*P*)^2^ + 0.0493*P*] where *P* = (*F*_o_^2^ + 2*F*_c_^2^)/3
965 reflections	(Δ/σ)_max_ < 0.001
47 parameters	Δρ_max_ = 0.62 e Å^−^^3^
0 restraints	Δρ_min_ = −0.34 e Å^−^^3^

### Special details

**Table:**

Geometry. All e.s.d.'s (except the e.s.d. in the dihedral angle between two l.s. planes) are estimated using the full covariance matrix. The cell e.s.d.'s are taken into account individually in the estimation of e.s.d.'s in distances, angles and torsion angles; correlations between e.s.d.'s in cell parameters are only used when they are defined by crystal symmetry. An approximate (isotropic) treatment of cell e.s.d.'s is used for estimating e.s.d.'s involving l.s. planes.
Refinement. Refinement of *F*^2^ against ALL reflections. The weighted *R*-factor *wR* and goodness of fit *S* are based on *F*^2^, conventional *R*-factors *R* are based on *F*, with *F* set to zero for negative *F*^2^. The threshold expression of *F*^2^ > σ(*F*^2^) is used only for calculating *R*-factors(gt) *etc*. and is not relevant to the choice of reflections for refinement. *R*-factors based on *F*^2^ are statistically about twice as large as those based on *F*, and *R*- factors based on ALL data will be even larger.Highest peak 0.62 at 0.4105 0.2353 0.4867 [0.74 A from C1] Deepest hole -0.34 at 0.1454 0.0185 0.3480 [0.59 A from N1]

### Fractional atomic coordinates and isotropic or equivalent isotropic displacement parameters (Å^2^)

**Table:**

	*x*	*y*	*z*	*U*_iso_*/*U*_eq_	
O1	−0.0014 (2)	0.11342 (10)	0.28187 (10)	0.0190 (3)	
N1	0.2436 (2)	0.05941 (11)	0.38743 (11)	0.0141 (3)	
C1	0.3636 (3)	0.31533 (12)	0.48341 (15)	0.0182 (3)	
H1A	0.1286	0.3338	0.4948	0.027*	
H1B	0.3910	0.3522	0.3832	0.027*	
H1C	0.5238	0.3680	0.5647	0.027*	
C2	0.4355 (3)	0.15172 (13)	0.49468 (13)	0.0145 (3)	
C3	0.6882 (3)	0.09028 (12)	0.60638 (14)	0.0146 (3)	
H3	0.8194	0.1543	0.6816	0.017*	

### Atomic displacement parameters (Å^2^)

**Table:**

	*U*^11^	*U*^22^	*U*^33^	*U*^12^	*U*^13^	*U*^23^
O1	0.0183 (4)	0.0186 (5)	0.0151 (5)	0.0029 (3)	−0.0081 (3)	0.0031 (3)
N1	0.0137 (4)	0.0150 (5)	0.0112 (5)	0.0004 (3)	−0.0028 (4)	0.0019 (4)
C1	0.0204 (5)	0.0122 (5)	0.0191 (6)	0.0012 (4)	−0.0022 (4)	0.0010 (4)
C2	0.0155 (5)	0.0136 (5)	0.0130 (5)	−0.0012 (4)	−0.0002 (4)	−0.0002 (4)
C3	0.0157 (5)	0.0134 (5)	0.0126 (5)	−0.0006 (4)	−0.0012 (4)	−0.0001 (4)

### Geometric parameters (Å, °)

**Table:**

O1—N1	1.2996 (12)	C1—H1B	0.9800
N1—C3^i^	1.3611 (14)	C1—H1C	0.9800
N1—C2	1.3681 (15)	C2—C3	1.3744 (15)
C1—C2	1.4863 (15)	C3—H3	0.9500
C1—H1A	0.9800		
			
O1—N1—C3^i^	120.44 (10)	H1B—C1—H1C	109.5
O1—N1—C2	120.62 (10)	N1—C2—C3	119.01 (10)
C3^i^—N1—C2	118.94 (10)	N1—C2—C1	118.14 (10)
C2—C1—H1A	109.5	C3—C2—C1	122.85 (10)
C2—C1—H1B	109.5	N1^i^—C3—C2	122.05 (10)
H1A—C1—H1B	109.5	N1^i^—C3—H3	119.0
C2—C1—H1C	109.5	C2—C3—H3	119.0
H1A—C1—H1C	109.5		

Symmetry codes: (i) −*x*+1, −*y*, −*z*+1.

### Hydrogen-bond geometry (Å, °)

**Table:**

*D*—H···*A*	*D*—H	H···*A*	*D*···*A*	*D*—H···*A*
C1—H1C···O1^ii^	0.98	2.41	3.3290 (15)	155
C3—H3···O1^ii^	0.95	2.31	3.1863 (15)	153

Symmetry codes: (ii) *x*+1, −*y*+1/2, *z*+1/2.
